# Genetic forms and pathophysiology of essential arterial hypertension in minor indigenous peoples of Russia

**DOI:** 10.1186/s12872-020-01464-7

**Published:** 2020-04-15

**Authors:** Tatyana Mulerova, Evgenya Uchasova, Michael Ogarkov, Olga Barbarash

**Affiliations:** Federal State Budgetary Institution “Research Institute for Complex Issues of Cardiovascular Diseases”, 6, Sosnoviy Blvd, 650002 Kemerovo, Russia

**Keywords:** Arterial hypertension, Pathophysiology, Genetic forms, Plasma renin activity, Ethnic groups

## Abstract

**Background:**

To study the genetic forms and pathophysiology of arterial hypertension by evaluating plasma renin activity in the Shors, minor indigenous peoples inhabiting the south of Western Siberia.

**Methods:**

A single-stage study of indigenous (the Shors) and non-indigenous peoples living in the villages of Gornaya Shoria of the Kemerovo region in the south of Western Siberia was conducted in the period from 2013 to 2017. One thousand four hundred nine adults (901 Shors and 508 non-indigenous inhabitants) were recruited in the study using a continuous sampling plan. Arterial blood pressure was measured according to 2018 ESC/ESH guidelines for the management of arterial hypertension. All the respondents underwent clinical and instrumental examination. Plasma renin activity was determined by enzyme-linked immunoassay with the BRG kits (Germany). Polymorphisms of *ACE* (I/D, rs 4340), *АGT* (c.803 T > C, rs699), *AGTR1* (А1166С, rs5186), *ADRB1* (с.145A > G, Ser49Gly, rs1801252) and *ADRA2B* (I/D, rs 28,365,031) genes were tested using polymerase chain reaction.

**Results:**

Renin-dependent hypertensive patients prevailed in both ethnic groups (65.6% in the indigenous group vs. 89.8% in the non-indigenous group, *p* = 0.001). Prevalence of a volume-dependent AH was low in both groups (34.4% in the indigenous group vs. 10.2% in the non-indigenous group, *р* = 0.001). The D/D and Т/Т genotypes of the АСЕ [OR = 6.97; 95% CI (1.07–55.58)] and AGT [OR = 3.53; 95% CI (1.02–12.91)] genes were associated with the renin-dependent AH in the Shors. The C/C genotype of AGTR1 gene was found to predispose to the volume-dependent AH [OR = 5.25; 95% CI (1.03–27.89)]. The C/C genotype of AGTR1 gene was associated with moderate or high renin levels suggesting essential AH in the non-indigenous group [OR = 5.00; 95% CI (1.21–22.30), *р* = 0.029].

**Conclusion:**

An in-depth understanding of AH pathophysiology and its genetic forms ensures the optimal choice of blood pressure-lowering treatment and optimizes AH control.

## Background

Essential arterial hypertension (AH) is one of the most prevalent modifiable cardiovascular risk factor worldwide. Its monitoring and effective control is a global goal focused on reducing morbidity and mortality of cardiovascular diseases [1].

The interaction of the sympathoadrenal system (SAS) and the renin-angiotensin-aldosterone system (RAAS) plays a major role in blood pressure (BP) control in the human body. Both, RAAS and SAS, are multifunctional players in AH that are closely related with each other. Increased SAS activity enhances the renin synthesis in the juxtaglomerular cells through renal sympathetic nerves activated by intrarenal β1-adrenoreceptors and circulating catecholamine stimulation [2]. Renin is a proteolytic enzyme that catalyzes the first step in the activation of the RAAS cascade and the subsequent production of angiotensin II, the main effector molecule of the RAAS [3].

Systemic RAAS activation occurs with elevated plasma renin activity (PRA) which is generally considered to reflect the level of renin activity [4]. It has a direct vasoconstrictive effect that may promote the development of AH associated with medium to high renin levels (a renin-dependent AH). However, low levels of renin may be also associated with AH. An increased salt intake induces a rise in plasma sodium leading an increase in extracellular fluid volume and resulting in a volume-dependent AH [5].

The genetic contribution to the onset of arterial hypertension have been recently discussed. Extensive research has been performed on the analysis of pathophysiological mechanisms, gene polymorphisms and gene expression levels associated with increased BP. Current evidence suggests that gene polymorphisms encoding components of the RAAS play an important role in the onset of essential AH. Angiotensin converting enzyme is a central component of the RAAS. The ACE gene is located on long arm of chromosome 17 (17q23). More than two dozen ACE gene polymorphisms have been reported so far. However, the I/D polymorphism which is distinguished by the insertion or deletion of an Alu element within intron 16 is considered as the most significant. The angiotensinogen gene (*AGT*) is located on chromosome 1 q42–43. Several AGT polymorphisms have been described, particularly that with the thymine nucleotide (T) replaced with cytosine (C) in the coding region of the gene (c.803 T > C, rs699). It leads to the substitution of threonine to methionine at position 235 (Met235Thr). This substitution changes the properties of angiotensinogen. Angiotensinogen II receptor type 1 (*AGTR1*) plays an important role in the pathogenesis of AH. It is located on long arm of chromosome 3 (3 g21–25). About 20 polymorphisms of this gene have been described. The most well studied AGTR1 SNP is rs5186 (also referred as A1166C). It has been previously shown that a single-nucleotide polymorphism in the 3′ untranslated region leads to the transversion of adenine to cytosine at position 1166. The effects of SAS on the cardiovascular system is mediated through adrenoceptors. The chronotropic and inotropic effects of the SAS on the cardiac function are manifested through β1- adrenoceptors. The role of two major genes (*ADRB1* and *ADRA2B*) in the development of hypertension has been also discussed.

Hence, candidate genes play a special role in the pathogenesis of AH. They determine the synthesis of angiotensin-converting enzyme (*АСЕ*), angiotensinogen (*AGT*), angiotensinogen II receptor type 1 (*AGTR1*), β1-adrenoreceptor (*ADRB1*), α2β-adrenoreceptor (*ADRA2B*) [6, 7]. These gene products are involved in the regulation of BP, electrolyte and fluid balance and the activation of sympathetic nervous system and functional capacities of the body. Nevertheless, the effects of polymorphisms in candidate genes on the levels of circulating factors of the RAAS and predominantly PRA remain unclear [8]. They are of particular concern for minor indigenous peoples that might have slightly different physiological mechanisms of adaptation, morphological and functional features.

Thus, measurements of PRA coupled with a comprehensive assessment of the genetic contribution to the onset of essential AH may provide new insights into the understanding of the interaction of these mechanisms and their role in the disease onset.

### Purpose

Our study was aimed at evaluating the genetic contribution to the onset of pathophysiological arterial hypertension by measuring plasma renin activity in the Shors, minor indigenous peoples living in the south of Western Siberia.

## Methods

A single-stage study of indigenous (the Shors) and non-indigenous population living in the villages of Gornaya Shoria of the Kemerovo region in the south of Western Siberia was performed in the period from 2013 to 2017. The Shors are minor indigenous peoples of the South-Siberian Mongoloid race. Using a continuous sampling method, 1409 adults (901 Shors and 508 non-indigenous inhabitants) were recruited into the study. The ethnic groups were comparable of gender and age. The recruitment of study participants was supported and facilitated by the local government. All participants gave written informed consent in accordance with the declaration of Helsinki. Respondents participating in the study were fully informed about the research objectives. Each participant received a unique identification number. The study design was approved by the Local Ethics Committee of the Federal State Budgetary Institution “Research Institute for Complex Issues of Cardiovascular Diseases” (Kemerovo, Russian Federation). The clinical examination included medical history review and collection, physical exam and a visit to a cardiologist.

Arterial blood pressure was measured in accordance with 2018 ESC/ESH guidelines for the management of arterial hypertension by an Omron automatic tonometer (Japan). Arterial hypertension was defined as a systolic blood pressure of 140 mmHg or more, a diastolic blood pressure of 90 mmHg or more, and / or taking antihypertensive medication. Patients with AH were allocated from each ethnic cohort (367 participants (40.7%) among the Shors and 230 subjects (45.3%) among non-indigenous population). Two hundred fifty-five participants (69.5%) out of 367 Shors did not take any antihypertensive medication just as 115 non-indigenous participants (50.0%). Main clinical and demographic data of hypertensive and non-hypertensive participants depending on the ethnic group are presented in Tables 1 and 2.

**Table 1:** Characteristic of patients with AH among the population of Mountain Shoria taking into account the ethnic peculiarities

Indicator	Indigenous population			Non-indigenous population			Р (1 VS 3)
	AH	Without AH	р	AH	Without AH	р	
	1	2		3	4		
Age, years, M ± SD	59.0 ± 12.3	42.3 ± 13.4	0.000	59.4 ± 11.6	41.5 ± 14.5	0.000	0.700
Height, cm, M ± SD	155.3 ± 7.7	157.7 ± 8.2	0.000	161.3 ± 9.5	163.9 ± 8.4	0.001	0.0001
Weight, kg, M ± SD	64.8 ± 13.5	60.2 ± 12.7	0.000	80.5 ± 18.0	72.0 ± 20.6	0.000	0.0001
Smoking, n(%)	95(25.9)	201(37.6)	0.001	53(23.0)	113(40.7)	0.000	0.434
BMI, kg/m^2^, M ± SD	26.9 ± 5.5	24.2 ± 4.7	0.000	30.9 ± 6,4	27.1 ± 6.3	0.000	0.0001
BMI ≥30 kg/m^2^, n(%)	101(27.5)	61(11.4)	0.000	123(53.5)	72(25.9)	0.000	0.0001
WC, cm, M ± SD	88.0 ± 12.2	80.3 ± 11.4	0.000	97.6 ± 15.2	85.0 ± 14.7	0.000	0.0001
WC ≥80 cm in women and ≥ 94 cm in men, n(%)	136(37.1)	102(19.1)	0.000	135(58.7)	82(29.5)	0.000	0.0001
SBP, mmHg, M ± SD	158.1 ± 20.4	117.7 ± 11.6	0.000	150.3 ± 19.0	119.7 ± 10.7	0.000	0.0001
DBP, mmHg, M ± SD	93.0 ± 11.0	75.4 ± 7.3	0.000	90.2 ± 12.99	76.2 ± 7.2	0.000	0.004
TC, mmol/l, M ± SD	5.83 ± 1.26	5.16 ± 1.21	0.000	5.89 ± 1.35	5.20 ± 1.29	0.000	0.583
TC, mmol/l, n(%)	243(75.5)	230(51.7)	0.000	163(79.5)	127(58.3)	0.000	0.282
LDL-C, mmol/l, M ± SD	3.50 ± 1.08	2.93 ± 1.05	0.000	3.53 ± 1.02	3.18 ± 1.22	0.004	0.766
LDL-C > 3.0 mmol/l, n(%)	175(63.2)	141(40.1)	0.000	124(70.5)	83(49.7)	0.001	0.111
HDL-C, mmol/l, M ± SD	1.42 ± 0.52	1.45 ± 0.54	0.404	1.22 ± 0.35	1.30 ± 0.41	0.054	0.0001
HDL-C < 1.2 mmol/l in women, < 1.0 mmol/l in men, n(%)	76(27.5)	78(22.4)	0.135	79(45.1)	56(33.9)	0.035	0.0001
TG, mmol/l, M ± SD	1.70 ± 1.44	1.36 ± 0.95	0.000	2.23 ± 1.49	1.70 ± 1.23	0.000	0.0001
TG > 1.7 mmol/l, n(%)	103(31.9)	99(22.3)	0.003	113(54.9)	75(34.4)	0.001	0.0001
Glucose, mmol/ l, M ± SD	5.86 ± 1.77	5.35 ± 0.94	0.000	6.14 ± 1.89	5.31 ± 0.82	0.000	0.108

*BMI* Body mass index, *WC* Waist circumference, *SBP* Systolic blood pressure, *DBP* Diastolic blood pressure, *TC* Total cholesterol, *LDL-C* Low-density lipoprotein cholesterol, *HDL-C* High-density lipoprotein cholesterol, *TG* Triglycerides

**Table 2:** Characteristic of patients with R-D AH and V-D AH among the population of Mountain Shoria taking into account the ethnic peculiarities

Indicator	R-D AH			V-D AH		
	Indigenous population	Non-indigenous population	р	Indigenous population	Non-indigenous population	р
Age, years, M ± SD	55.8 ± 14.0	60.3 ± 11.4	0.003	61.9 ± 11.7	58.4 ± 12.1	0.089
Height, cm, M ± SD	156.0 ± 7.4	160.6 ± 9.2	0.000	154.8 ± 7.8	162.2 ± 9.8	0.000
Weight, kg, M ± SD	64.9 ± 13.2	81.2 ± 17.2	0.000	64.8 ± 13.6	79.7 ± 20.0	0.000
Smoking, %	30.5	22.3	0.088	23.9	25.3	0.791
BMI, kg/m^2^, M ± SD	26.7 ± 5.4	31.5 ± 6.2	0.000	27.2 ± 5.6	30.3 ± 7.1	0.000
BMI ≥30 kg/m^2^, %	24.6	56.6	0.000	28.9	48.0	0.002
WC, cm, M ± SD	89.2 ± 12.1	99.4 ± 14.9	0.000	87.3 ± 12.2	94.5 ± 15.3	0.000
WC ≥80 cm in women and ≥ 94 cm in men, %	43.0	63.3	0.000	34.2	50.7	0.010
SBP, mmHg, M ± SD	154.6 ± 20.1	147.8 ± 20.8	0.038	162.7 ± 20.5	152.4 ± 16.5	0.000
DBP, mmHg, M ± SD	90.3 ± 11.4	88.6 ± 13.8	0.292	95.9 ± 10.8	91.4 ± 11.7	0.044
ТС, ммоль/л, M ± SD	5.70 ± 1.16	5.93 ± 1.46	0.182	5.88 ± 1.30	5.79 ± 1.13	0.101
TC, mmol/l, %	73.4	78.9	0.191	77.7	79.5	0.627
LDL-C, mmol/l, M ± SD	3.45 ± 1.10	3.59 ± 1.08	0.284	3.55 ± 1.07	3.47 ± 0.83	0.344
LDL-C > 3.0 mmol/l, %	58.3	70.8	0.056	66.1	69.9	0.920
HDL-C, mmol/l, M ± SD	1.39 ± 0.53	1.24 ± 0.37	0.008	1.44 ± 0.51	1.18 ± 0.26	0.002
HDL-C < 1.2 mmol/l in women, < 1.0 mmol/l in men, %	32.3	44.4	0.059	24.9	46.8	0.004
TG, mmol/l, M ± SD	1.69 ± 1.22	2.23 ± 1.47	0.010	1.71 ± 1.52	2.23 ± 1.48	0.014
TG > 1.7 mmol/l, %	32.9	53.4	0.000	31.0	55.5	0.000
Glucose, mmol/l, M ± SD	5.66 ± 1.63	6.32 ± 2.16	0.028	5.92 ± 1.81	5.79 ± 0.92	0.425

*V-D AH* Volume-dependent arterial hypertension, *R-D AH* Renin-dependent arterial hypertension, *BMI* Body mass index, *WC* Waist circumference, *SBP* Systolic blood pressure, *DBP* Diastolic blood pressure, *TC* Total cholesterol, *LDL-C* Low-density lipoprotein cholesterol, *HDL-C* High-density lipoprotein cholesterol, *TG* Triglycerides

PRA was measured in all participants who did not receive any antihypertensive medication (255 Shors participants and 115 non-indigenous participants). Of them, 1.6% of the Shors and 5.8% of non-indigenous participants had the estimated glomerular filtration rate below 60 ml/min/1.73 m^2^, 21.0 and 30.1% - below 90 ml/min/1.73 m^2^, and 77.4 and 64.1% - ≥ 90 ml/min/1.73 m^2^, respectively.

The morning after an overnight fast, venous blood samples were withdrawn from the cubital vein in all patients. Blood samples were centrifuged; serum was frozen and banked for analysis at deep freeze temperature. Samples were delivered to the laboratory in the liquid nitrogen containers avoiding thawing. PRA was measured by enzyme-linked immunoassay using the BRG kits (Germany). The upper limit of the reference range of renin level in healthy people in an upright position is 47.85 pg/ml. Therefore, all hypertensive participants were subdivided into 2 groups. Patients with plasma renin levels over 47.85 pg/ml were assigned to Group 1 and patients with plasma renin levels below or equal to 47.85 pg/ml were assigned to Group 2. The onset of AH in Group 1 patients was associated with the excessive renin release and the subsequent RAAS activation, whereas in Group 2 patients it was associated with the suppression of renin release and sodium retention in the body.

Polymorphisms of *ACE* (I/D, rs4340), *АGT* (c.803 T > C, rs699), *AGTR1* (А1166С, rs5186), *ADRB1* (с.145A > G, Ser49Gly, rs1801252) and *ADRA2B* (I/D, rs28365031) genes were tested using polymerase chain reaction. The detailed description of the laboratory protocol was previously described in [9].

The statistical analysis was performed using the commercially available Statistica 6.0 (StatSoft Inc., USA) software. The normality of the distribution was tested using the Kolmogorov-Smirnov test. Continuous variables are presented as the mean and the standard deviation (M ± SD). Categorical variables are presented as the frequencies or the percentages. For categorical variables, the weights were calculated to eliminate any possible biases. To estimate the statistical significance of the differences between categorical variables, contingency tables were generated with the subsequent calculation of Pearson’s chi-squared test. Logistic regression adjusted to the age and gender was used to determine the presence of any relationships between the genetic factors and pathophysiological forms of AH. Odds ratios (OR) and 95% confidence intervals (CI) were estimated.

## Results

The overall prevalence of AH in Gornaya Shoria was 42.4%. Out of 42.4, 40.7% cases were found among the Shors and 45.3% among the non-indigenous participants (*p* = 0.098). The renin-dependent AH was more commonly found in both ethnic groups as compared to the volume-dependent AH (65.6% vs 34.4% (*p* = 0.001) among the indigenous group and 89.8% vs 10.2% (*p* = 0.001) among the non-indigenous group).

The mean renin level was lower (86.1 ± 65.4 pg/ml) in the hypertensive Shors than that in non-indigenous participants (115.9 ± 73.9 pg/ml, *p* = 0.0001). In addition, the defined differences between the ethnic groups in the mean renin values were consistent with the differences of the polymorphisms in candidate genes encoding the components of the RAAS and SAS. Thus, the Shors who were the carriers of the I/D, А/А and D/D genotypes of the *АСЕ*, *ADRB1* and *ADRA2B* genes had lower renin levels (73.9 ± 53.0 pg/ml, 80.7 ± 52.3 pg/ml and 99.2 ± 64.6 pg/ml), compared with non-indigenous participants who had the same genotypes – 111.6 ± 71.0 pg/ml (*р* = 0.010), 116.1 ± 65.3 pg/ml (*р* = 0.004) and 161.5 ± 89.4 pg/ml (*p* = 0.0001), respectively. Moreover, the D/D genotype of *АСЕ* gene among the Shors was associated with the highest renin levels (125.0 ± 71.3 pg/ml) as compared to those who were the carriers of the I/D genotype (73.9 ± 53.0 pg/ml, *р* = 0.004) and the I/I genotype (87.1 ± 57.0 pg/ml, *р* = 0.030). A similar pattern was determined for ADRA2B gene polymorphism: the indigenous participants who were the carriers of the minor D allele in the homozygous state had a higher renin level as compared to participants with the I/D genotype (99.2 ± 64.6 pg/ml vs. 62.3 ± 30.4 pg/ml, *p* = 0.011). Non-indigenous participants carrying the homozygous D/D genotype of *ADRA2B* gene demonstrated a higher renin level as compared to the carriers of the I/I genotype (161.5 ± 89.4 pg/ml vs. 106.7 ± 58.0 pg/ml, respectively, *р* = 0.047).

Table 3 summarizes the frequency distribution of the genotypes of candidate genes encoding the components of the RAAS and SAS among participant from different ethnic groups. Renin-dependent and volume-dependent AH were encoded by different genes based on participants’ ethnicity.

**Table 3:** Genotype frequency of candidate genes (*АСЕ, AGT, AGTR1, ADRB1, ADRA2B*) in the indigenous population and non-indigenous population of Mountain Shoria with various pathophysiological forms of AH

Genotype	Indigenous population			Non-indigenous population		
	V-D AH	R-D AH	р	V-D AH	R-D AH	р
Gene *АСЕ*, rs4340						
I/I	44.4	42.3	0.719	22.2	29.6	0.534
I/D	51.8	36.6	0.130	44.4	53.7	0.829
D/D	3.8	21.1	0.035	33.3	16.7	0.321
Gene *AGT*, rs699						
T/T	46.9	20.0	0.046	45.4	47.0	0.812
T/C	31.2	40.0	0.480	27.3	17.7	0.283
C/C	21.9	40.0	0.177	27.3	35.3	0.529
Gene *AGTR1*, rs5186						
A/A	65.6	55.6	0.426	35.7	19.2	0.229
A/C	28.1	18.5	0.261	42.9	23.1	0.112
C/C	6.3	25.9	0.041	21.4	57.7	0.029
Gene *ADRB1*, rs1801252						
A/A	59.2	63.0	0.621	50.0	72.9	0.108
A/G	33.8	25.9	0.371	50.0	22.9	0.059
G/G	7.0	11.1	0.520	0.0	4.2	0.582
Gene *ADRA2B,* rs28365031						
I/I	23.9	50.0	0.115	29.6	50.0	0.104
I/D	53.5	31.3	0.163	37.0	31.3	0.886
D/D	22.6	18.7	0.287	33.4	18.7	0.098

*V-D AH* Volume-dependent arterial hypertension, *R-D AH* Renin-dependent arterial hypertension

The frequency of the D/D genotype of *АСЕ* gene prevailed among the Shors with renin-dependent AH than among the Shors with volume-dependent AH (21.1% vs. 3.8%, *p* = 0.035)., The Т/Т genotype (rs699) of *AGT* gene was commonly found among hypertensive participants with increased renin levels than among participants with low-renin AH (46.9% vs. 20.0%, *р* = 0.046). Thus, the D/D and Т/Т genotypes of *АСЕ* and *AGT* genes were associated with the renin-dependent AH in the indigenous group [OR = 6.97; 95% CI (1.07–55.58)] and [OR = 3.53; 95% CI (1.02–12.91)]. The minor C/C genotype of *AGTR1* gene was associated with the volume-dependent AH. The odds ratios increased 5-fold to determine the volume-dependent AH in the C/C genotype carriers as compared to the A/A and A/C genotype carriers [OR = 5.25; 95% CI (1.03–27.89)]. The prevalence of the mutant allele C in the homozygous state among volume-dependent AH participants was 25.9 and 6.3% among renin-dependent AH participants (*р* = 0.041).

The presence of *AGTR1* gene polymorphism was associated with the onset of AH in the non-indigenous group. The frequency of C/C genotype of this gene was higher among patients with renin-dependent AH (57.7%) as compared to patients with volume-dependent AH (21.4%) [OR = 5.00; 95% CI (1.21–22.30), *p* = 0.029].

## Discussion

Measuring PRA gives an opportunity to determine one of two underlying mechanisms responsible for the onset of AH [3]. Egan et al. (2009) have previously demonstrated the significance of PRA estimation in patients with AH [10]. Unfortunately, this method has not got a wide application in the Russian Federation.

Our data from the epidemiological study in Gronaya Shoria are consistent with recent evidences that have proved that two-thirds of patients have renin-dependent AH, while the others are present with volume-dependent AH [11, 12]. Renin-dependent AH prevailed in both ethnic groups. Given similar prevalences of AH in the study groups, the mean renin level was lower among the Shors than among non-indigenous participants. This pattern may be explained by the presence of other mechanisms increasing blood pressure among minor indigenous peoples in addition to the RAAS and SAS. The Shors consume more alcohol, which is considered as a risk factor for hypertension. The indigenous group consumed more alcohol than the non-indigenous group (43.5% vs. 18.8%, *p* = 0.005). The Shors people are hunter-gatherers who spend a lot of time in the Taiga (so-called Russian forest in Siberia) eating food with excessive salt content. This nutrition pattern contributes to the elevation of blood pressure. Our data were consistent with the data reported by Akintunde et al. (2018), who found that the Caucasians had a higher renin activity as compared to people from other ethnic groups [13].

The genetic contribution to the onset of AH is beyond doubt [14]. However, the studies describing the effects of the alleles of the candidate genes of the RAAS and SAS on the development of various pathophysiological types of AH are still limited. In 1999, Francke et al. [15] focused at assessing the presence of the associations between the polymorphisms of 5 genes involved in AH pathogenesis (angiotensin converting enzyme, kallikrein, angiotensinogen, angiotensin II type I receptor and renin) and salt sensitivity. The obtained data suggested that the volume-dependent AH was associated with the I/D polymorphism of *АСЕ* gene (D-allele). Kato et al. and Giner et al. determined the association of the *АСЕ* gene with BP increase in response to salt intake in patients with AH [16, 17]. The study on the Japanese cohort reported the presence of the association between the onset of the volume-dependent AH and the I/I genotype of the *ACE* gene [18]. In our study, we found the association of the D/D genotype of *АСЕ* gene with renin-dependent AH in the Shors. Therefore, the treatment effectiveness for hypertensive patients carrying the mutant allele D may be optimized through the medications inhibiting the RAAS (angiotensin-converting enzyme inhibitors, angiotensin receptor blockers, direct renin inhibitors).

We have previously shown that the minor allele C of *AGTR1* gene was associated with AH in both ethnic groups [19]. Many studies have demonstrated a positive association of the C allele of *AGTR1* gene with AH [20–22]. A recent meta-analysis of published studies with 28,952 patients allowed identifying the association between the polymorphism of *AGTR1* gene with the increased risk of AH in the Asians and the Caucasians [22]. The carriers of C/C genotype among the Egyptians showed more severe complications of this disease [23]. However, there are no data suggesting the relationship of the polymorphism of this gene with PRA. In our study, we identified that the C/C genotype was associated with the development of various pathophysiological forms of AH in the individuals from different ethnic groups. The C/C genotype of *AGTR1* in the Shors was associated with the development of the volume-dependent AH, whereas in the non-indigenous group with the renin-dependent AH.

## Conclusion

The measurement of PRA plays the key role in the evaluation of AH underlying pathology based on the understanding of the interaction between the RAAS and the mechanism of maintaining the fluid balance in the body through kidney sodium reabsorption and fluid retention. Our results allow identifying the genetic markers indicative for pathophysiological variant of AH. The D/D and T/T genotypes of the *ACE* and *AGT* candidate genes were associated with renin-dependent AH and the С/С genotype of *AGTR1* gene with the volume-dependent AH in the Shors. Alternatively, the C/С genotype of *AGTR1* gene was associated with the renin-dependent AH in non-indigenous participants. An in-depth understanding of the pathophysiological mechanism underlying AH and genetic contribution may facilitate the choice of antihypertensive medication and optimize the treatment of this disease.

## Data Availability

The Ethics Committee restricts access to the original patient database that requests can be directed to them. Although the database is not available, we can provide anonymous patient data at a reasonable to the appropriate author.

## Abbreviations

*АСЕ*: Angiotensin-converting enzyme candidate gene
*ADRA2B*: α2β-adrenoreceptor candidate gene
*ADRB1*: β1-adrenoreceptor candidate gene
*AGT*: Hypertensinogen candidate gene
*AGTR1*: Angiotensinogen II receptor type 1 candidate gene
AH: Arterial hypertension
BMI: Body mass index
BP: Blood pressure
CI: Confidence interval
DBP: Diastolic blood pressure
HDL-C: High-density lipoprotein cholesterol;
LDL-C: Low-density lipoprotein cholesterol
OR: Odds ratio
PRA: Plasma renin activity
RAAS: Renin-angiotensin-aldosterone system
R-D AH: Renin-dependent arterial hypertension
SAS: Sympathoadrenal system
TC: Total cholesterol
SBP: Systolic blood pressure
TG: Triglycerides
V-D AH: Volume-dependent arterial hypertension
WC: Waist circumference
